# Inverting the logic in amyotrophic lateral sclerosis

**DOI:** 10.1093/brain/awaf276

**Published:** 2025-07-30

**Authors:** Martin R Turner

**Affiliations:** Nuffield Department of Clinical Neurosciences, University of Oxford, John Radcliffe Hospital, Oxford OX3 9DU, UK

## Abstract

Turner highlights survivorship bias in the study of neurodegenerative disorders such as ALS, suggesting that meaningful progress may require a fundamental shift in focus from the affected to the resilient, and from what is seen to what is not. Neurodegeneration should be viewed as a loss of tolerance as much as a gain of toxicity.

***The suggestion is that meaningful progress in neurodegenerative disorders requires a fundamental shift in focus from the affected to the resilient. This perspective needs to be implemented across genetics, proteins, cells, tracts and networks, as well as populations. Secondly, neurodegenerative disorders need to be explored as representing a loss of tolerance as much as a gain of toxicity.***

Though possibly containing apocryphal elements, the story of mathematician Abraham Wald (1902–50) who served during World War II as part of the USA’s Statistical Research Group, provides a useful analogy. Wald’s group was tasked with reducing the loss of bombers to enemy attack over Europe. In the analysis of the regional patterns of damage to aircraft returning from these sorties, Wald is said to have made the pivotal ‘inversion of logic’ in recognizing that the distribution of damage in returning aircraft must be in non-critical areas. Those with damage elsewhere failed to return because critical areas had been hit and the hulls lost to observation. This concept of survivorship bias led to the selective reinforcement of undamaged rather than damaged areas (Fig. 1).

**Figure 1:**
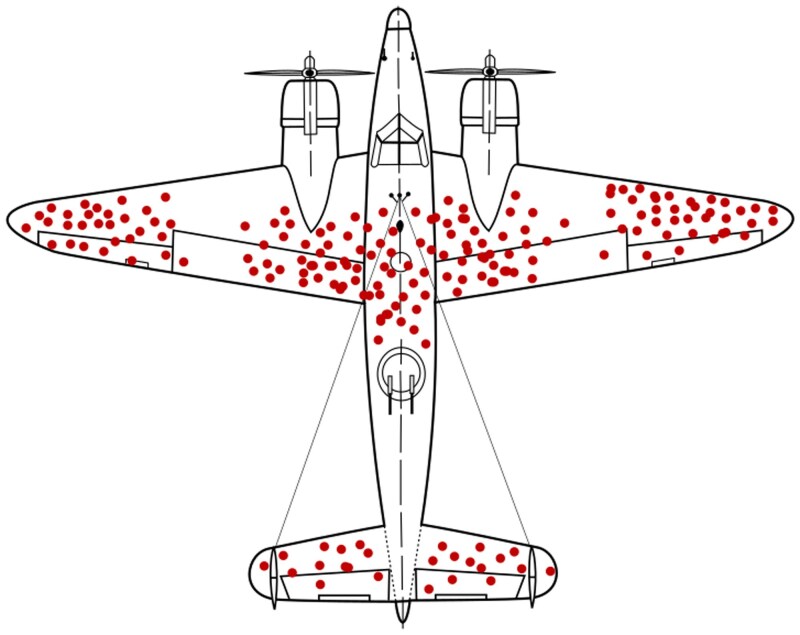
**Image of the distribution of damage to USA aircraft returning from bombing sorties in World War II.** The recognition of ‘survivorship bias’ prompted focus of reinforcement of the undamaged rather than the damaged areas. From https://en.wikipedia.org/wiki/Survivorship_bias (reproduced under a Creative Commons Licence).

The progressive loss of nervous system tissue and function, encompassed by the term neurodegeneration, is strongly associated with older age. The near doubling in human life expectancy over the past century has ‘revealed’ neurodegenerative disorders and placed them among the largest threats to global wellbeing, without this necessarily representing an acquired toxicity of a technological era. ‘A Lecture on Abiotrophy’ by pre-eminent neurologist Sir William Gowers (1845–1915) in 1902,^1^ might be considered a prototypical concept for the concept of neurodegeneration, arising as a loss of tolerance exposed by ageing. Gowers noted ‘a degeneration or decay in consequence of a defect of vital endurance’. The term abiotrophy appears to recognize a process distinct from the ‘traditional’ model of disease caused by toxic or infectious processes; one that might involve a more gradual deficit in homeostatic reserves.

## Clinical lumping and molecular splitting

Neurodegenerative syndromes were originally defined according to clinical phenotypes forged through the painstaking (often beautifully illustrated) observations of 19th century anatomists. For Jean-Martin Charcot (1825–93), his eponymous syndrome of amyotrophic lateral sclerosis (ALS) remains so characteristic in its core features to still comfortably represent one syndrome in the neurology clinic today. However, the classification of neurodegenerative disorders according to clinical phenotypes increasingly jars with the more complex contemporary taxonomy based upon molecular signatures. These signatures are typically built around a protein aggregate, but with limited correlation of nervous system regional location and clinical heterogeneity.

The post-mortem identification of ubiquitinated neuronal and glial cytoplasmic inclusions of nuclear-depleted TDP-43 in 97% of cases of ALS and 50% of frontotemporal dementia (FTD) cemented a pathological overlap that can be found in some of these much earlier clinical observations. This apparently unifying discovery increasingly collides with an expanding array of diverse upstream cellular pathway perturbations jostling for primacy in the pathogenesis of ALS, including: excitotoxicity, oxidative stress, mitochondrial dysfunction, axonal transport deficits, glial pathology, autophagy, nuclear-cytoplasmic transport and RNA biology.^2^ Primacy given to the canonical function of the gene associated with the protein aggregate, and an approach to pathogenesis and spread derived from end-stage post-mortem tissue, risks the survivorship bias highlighted in the story of Abraham Wald.

## A need for small beginnings

The identification of monogenetic variants associated with the common neurodegenerative phenotypes means that the challenge with any putative core pathomechanism is to understand how it is subtle enough to accommodate the latency in symptom onset. This is challenging to construct when major perturbations in fundamental cellular functions are implicated, such as aberrant nuclear cytoplasmic transport or cellular biophysics such as liquid-liquid phase separation.

Across Alzheimer’s disease, Parkinson’s disease and ALS, pathological variants in the single gene associated with the predominant protein aggregate consistently account for a very small proportion of cases. The physiological role for the genes has proved challenging to link simplistically with the histopathologically-derived pathogenic narrative. This is unsurprising if such genes have independent roles in neurodevelopment, adverse effects becoming apparent through a more complex interplay of factors visible only because of more routine human ageing. TDP-43 is increasingly seen as a potential therapeutic target in ALS, but its biology is complex,^3^ involving the regulation of thousands of human genes, with normal activity not physically confined to the nucleus. The post-mortem histopathological focus on its nuclear depletion as key event (confused by the term ‘mislocalization’) risks engendering a simplistic therapeutic strategy. Furthermore, pathological variants in the parent gene *TARDBP* (accounting for less than 0.5% of cases of ALS) appear tolerated for decades before the onset of symptoms. This might imply that these rare variants do not result in an early loss of canonical functions, interacting instead with more subtle dysfunction triggered in later life, with independent complex genetic determinants. Most recently, cryptic exon expression has been linked to loss of TDP-43 function in the symptomatic phase of ALS.^4^ An important question might be whether cryptic expression occurs from conception in *TARDBP* pathological variant carriers and what this implies if not.

Similarly, the hexanucleotide repeat expansion associated with *C9orf72*—associated with ∼10% of all cases of ALS (and FTD) in European and North American populations—is expressed in many body cell types, yet fundamental aspects of RNA biology dysfunction are implicated in theories of ALS pathogenesis. Even accepting a high level of functional redundancy in the nervous system, how impaired RNA biology is compatible with embryonic development, and a focus on the nature of any mitigation is vital to understand. A wider question is the phylogenetic origins of such repeat sequences, which occur throughout the human genome and may have currently obscure homeostatic roles that can deteriorate independently in age-related processes.

The analysis of incidence versus age of onset has been used to support a multi-hit model of pathogenesis in ALS,^5^ in which the common monogenetic causes are presumed to count towards one or more of the factors associated with the development of disease. Understanding the precise underpinnings of the variable penetrance of the common dominantly inherited causes of neurodegenerative disorders, will be essential to maximizing the study of those presymptomatic individuals at high risk of later disease through carriage of pathological genetic variants. There is a recognition of ascertainment bias in calculating penetrance, with typically higher estimates in pedigrees identified through ALS clinics, versus carrier data derived from wider population studies. The presence of a larger-than-expected number of asymptomatic, elderly carriers of pathogenic variants sampled across large-scale populations, might usefully prompt a pivot toward the study of the protective, ‘hero’ genetic variants, away from the hitherto focus on the ‘villains’. Epigenetic and environmental factors will have to be considered at the population scale, while somatic mutation challenges the concept of a single, unchanging nuclear genome, and may offer novel age-related mechanisms for the genesis of neurodegeneration.

## The clinical inferences and importance of architecture

The awareness of motor weakness in ALS can be typically timed to a specific month by most of those affected, usually with a clear focus e.g. a limb or speech in 90% of cases. The site of initial symptom onset and the pattern of symptomatic weakness progression in limb-onset cases is not random, though subject to the limitations and confirmation biases of the clinical examination. Kinnier Wilson (1878–1937) specifically noted ‘the acuteness of many cases’, alongside the fact that ‘the lesions appear systematised’.^6^ A contemporary molecular interpretation envisages ‘seeding’ and ‘prion-like’ spread of misfolded protein pathology through anatomical pathways. However, much of the foundation for this model has been a ‘staging’ based on the spatial extent of cerebral protein aggregation post-mortem. The assumption that this reflects sequential spread during life currently lacks clinical data with no link to disease duration found in the study of ALS.^7^

The most accepted and well-published examples of apparent selective vulnerability in ALS are the relative sparing of oculomotor and motor neurons arising from Onuf’s nucleus, yet attempts to identify a molecular underpinning have failed. Speculatively, an alternative substrate underlying the relative resistance might lie within the circuitry (architectural) differences associated with normal function. This might include differences in the patterns of tonic and stimulatory neurophysiology currently hidden to analytical tools. Study of comparative motor system anatomy and evolutionary considerations in the selective vulnerability of the ALS-FTD syndrome offers clues,^8^ and highlights the need for a much deeper understanding of the uniquely complex ‘convergence and divergence’ of the human corticospinal tract,^9^ including greater consideration of the somatotopic organization of the spinal cord anterior horns which might underlie clinical heterogeneity as much as molecular factors. Broader hypotheses have emerged that later-life cerebral degenerative syndromes may reflect pre-symptomatic structural connections,^10^ one in which development defines degeneration. Anatomical factors may prove to underlie the relative dichotomy of an ALS versus FTD phenotype associated with the same hexanucleotide expansion in *C9orf72*.

## The biomarkers of compensation rather than degeneration

The variable penetrance of genetic pathological variants linked to ALS implicates an untapped heterogeneity arising from the mitigating processes that provide the tolerance over the decades before functional reserves begin to be depleted and symptoms later emerge. Acquired deficits or genetic factors underlying the depletion of compensatory factors might be as relevant as the induction or accumulation of toxic ones. Studies of asymptomatic carriers of pathological genetic variants associated with ALS demonstrate a rise in blood neurofilament light chain levels preceding the emergence of motor symptoms. This is compatible with a neurodegenerative tipping point, but with a preceding cellular and network milieu that was heavily compensated, the basis of which might contain the key to preventative as well as symptomatic therapeutic intervention.

**Figure 2:**
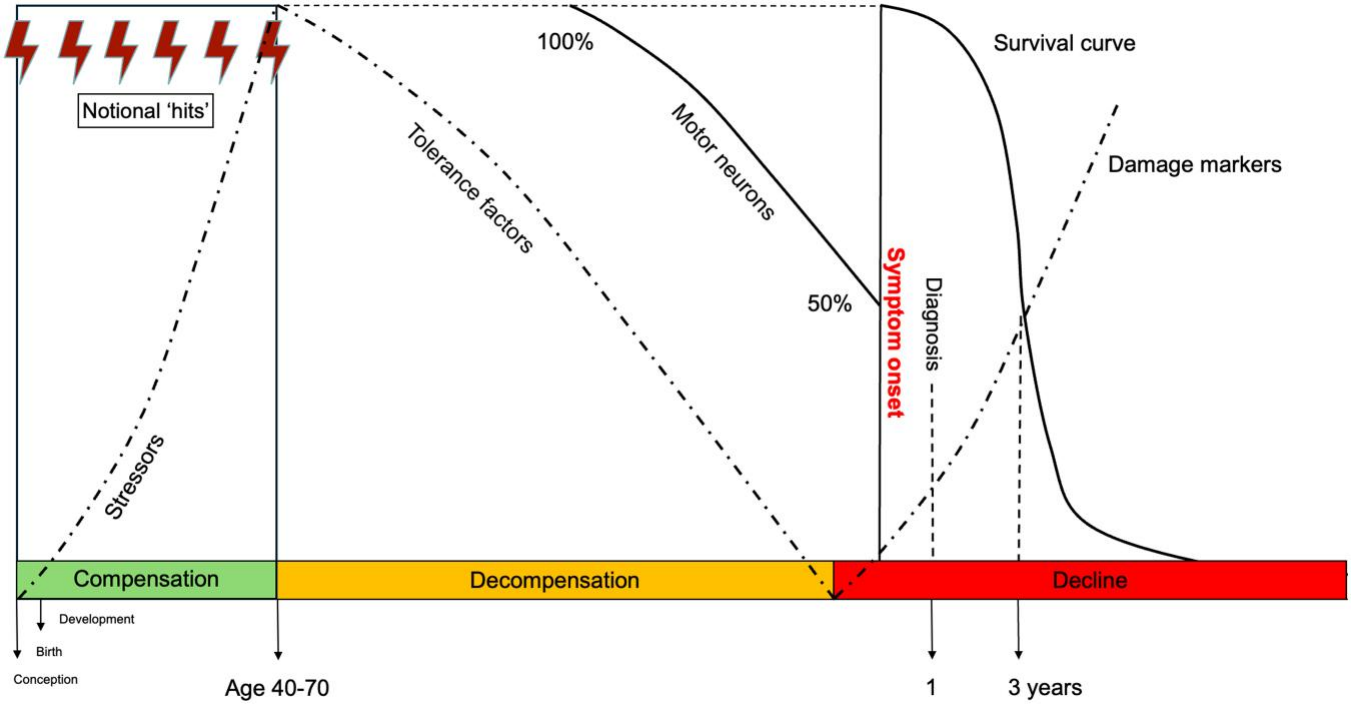
**A timeline for ALS divided into three phases.** In the ‘compensated’ phase from conception, rising stressors include the discrete steps within a multi-hit model, some of which are presumed to occur at conception for those with monogenetic pathological variants. The timing of the transition to the ‘decompensating’ phase coincides with a decline in the factors that have previously tolerated the stressors, and with independent genetic determinants. Nervous system redundancy suggests as much as 50% of motor neurons will be lost before symptoms appear, then marking the third phase. Here the biomarkers of damage begin to accumulate, including neurofilament light chain in the peri-symptomatic period. This final phase has hitherto been the source of biomarkers. Yet focus might be usefully also applied to the earlier markers of tolerance in search of effective therapy, which may not relate simply or at all to the pathways inferred from the biomarkers of loss. At present, this presymptomatic period is only accessible through the study of those at risk through monogenetic pathological variants and complicated by their variable penetrance. ALS = amyotrophic lateral sclerosis.

The recognition of symptoms as the late stage of a lifelong processes implies an earlier phase of cellular, biochemical tolerance, through compensation in the face of ‘hits’ (Fig. 2). The biomarkers of this compensated phase promise to unlock the strategies needed for prevention, but may be entirely distinct to biomarkers identified through the lens of the damage associated with the symptomatic stage. There is the potential for Waldian-type bias in the interpretation of molecular ‘-omics’ if the highest significance (particularly for drug targeting) is attributed to results related to pathways linked to the protein aggregate characterizing the end stage of disease.

## Asking the right questions

Neurodegenerative disorders have a common molecular theme of loss of protein homeostasis, for which brain health beyond the fourth decade of life may be particularly dependent and vulnerable. While aggregation is indisputably part of the accepted signature of such disorders, it is not necessarily the primary cause. Cells with insoluble aggregates are visible to histopathological study, but the cells which did not mount this response will have disintegrated without trace, like the aircraft lost to Abraham Wald’s observation. Without the option of a systematic study of brain tissue before death, it is currently difficult to explore the alternative logic of the nuclear depletion and aggregation of TDP-43 as part of an attempt to mitigate a wider independent set of stressors, or a physiological response to other distinct primary events in ALS pathogenesis.

There are important commonalities of the clinical syndromes that should not be overlooked, despite an expanding upstream network of diverse molecular changes (whose natural corollary envisages personalized medicine bewilderingly beyond any current model of drug therapy). A more agnostic approach to the interpretation of -omic studies is essential, not biased by focus only on the pathways linked to the symptomatic period, but open to pathways that reflect a presymptomatic state of compensation and tolerance. However, the window on presymptomatic events for a rare disease like ALS is currently only available through the study of carriers of pathological variants in single genes. Early events will have gene-specific upstream pathways, albeit still converging on common and characteristic clinical end-points, and influenced by independent modifying genetic factors underlying incomplete penetrance. A key part of the giant leap in understanding may come from the dedicated study of those who do not develop neurodegeneration. Bring on The Wellderly to answer the question: ‘Where did it all go right?’
